# Letter to the Editor RE: Assessing nonsexual transmission of the human papillomavirus: Do our current cleaning methods work? By Tucker et al.

**DOI:** 10.1002/jmv.28038

**Published:** 2022-08-08

**Authors:** Cori L. Ofstead, Abigail G. Smart

**Affiliations:** ^1^ Ofstead & Associates Inc. Bloomington Minnesota USA

To the Editor,

Tucker et al. concluded current endoscope reprocessing methods do not eliminate human papillomavirus (HPV) and may allow it to be transmitted from carriers to other otolaryngology clinic patients. The authors suggested that orthophthalaldehyde (OPA) high‐level disinfectant (HLD) “may not be fully effective at removing HPV from endoscopes.”^1^ We believe the evidence does not support their conclusions, and we are concerned that problems with study design and reprocessing quality may have contributed to erroneous conclusions.

In similar studies, researchers generally describe the endoscopes and provide information about factors impacting reprocessing effectiveness, such as endoscope age and condition. This article does not reveal the number or type of endoscopes sampled, and the authors assumed endoscopes acquired viral DNA during the most recent procedure or processing step. If the 24 samples were taken from fewer than 24 endoscopes, it is possible certain endoscopes persistently harbored viral DNA. The researchers did not describe steps taken to ensure endoscopes were free of viral DNA before study initiation, and, therefore, it is impossible to know whether viral DNA was already present. Moreover, they did not say how aseptic conditions were maintained during sampling. This is concerning because even minor deviations from prescribed practices^2^ can contaminate samples, and a recent study found that 41.6% had sampling errors.^3^

Investigations in other facilities utilizing OPA determined residual contamination was due to other causes. During an adenovirus outbreak among eight bronchoscopy patients, samples were taken from three bronchoscopes. Adenovirus DNA was detected in one, but there was no growth in viral cultures. Reprocessing was performed using an automated endoscope reprocessor (AER) with OPA by licensed personnel who had completed competency testing. The bronchoscopes underwent ethylene oxide sterilization before being sent to the manufacturer, which found critical defects requiring repair. Investigators deemed the cases pseudoinfections “resulting from nonviable adenovirus DNA remaining on bronchoscopes after cleaning and HLD,” potentially related to the use of damaged endoscopes and repeated bronchoscopy of an index patient with adenovirus pneumonia.^4^

Another outbreak investigation found bacterial pathogens in all samples taken from bronchoscopes that underwent manual HLD, but none in those reprocessed in an AER. Researchers sampled the sink, faucet, drainpipes, rinse water, reprocessing connecting tubes, and OPA. The outbreak strain was found in the rinse water, tubing, and sink drainpipes. No microorganisms were cultured from OPA. The contamination was terminated by replacing water filters, faucets, and plumbing.^5^ A review found it was nearly impossible to disinfect sinks and drains, and in most outbreaks, entire sinks and drain systems had to be replaced.^6^

Although current standards recommend endoscopes be sterilized or reprocessed using AERs to ensure HLD parameters are consistently met,^7^, ^8^, ^9^ Tucker et al. described using manual HLD—an outdated method. There was no indication the HLD container was cleaned and sterilized between uses, and endoscopes were rinsed after HLD by placing them in a sink filled with water. Although the sink was reportedly cleaned before rinsing, they did not mention disinfecting the sink or verifying it was free of viral DNA. This is troubling because post‐HLD rinses should be performed using sterile or highly filtered water,^7^, ^8^ and contaminated rinse water, sinks, and drains are well‐documented sources of pathogens that caused infection outbreaks.^5^, ^6^, ^10^

Studies have shown new endoscopes immersed in reprocessing sinks acquired protein, hemoglobin, and bacteria before clinical use could have introduced contaminants.^11^, ^12^ A multisite study detected the same high‐concern organisms on endoscopes and samples taken from sinks used for manual cleaning, rinse water, floors, and air near reprocessing areas.^3^ It seems possible that viral DNA found by Tucker et al. on endoscopes was introduced during immersion in a sink post‐HLD—a possibility that could have been ruled out if environmental samples had been tested.

There were no indications that other important aspects of standards were implemented in this clinic, such as performing leak tests and visual inspection, conducting tests to verify soil removal, and allowing only certified technicians to reprocess endoscopes.^7^, ^8^ The incorrect terminology used throughout the paper (e.g., referring to HLD as “cleaning”) is confusing and suggests the authors have a poor understanding of sterile processing. The apparent nonadherence with standards is concerning because even minor deviations can result in reprocessing failures.^7^, ^8^, ^9^ Given the apparent reprocessing deficiencies, there are too many unknowns to point a finger at the OPA chemistry used for the HLD step. More importantly, the presence of viral DNA on more endoscopes after processing than before processing suggests there are serious deficiencies in the processes used to ensure this facility's endoscopes are clean and free of defects or other pathogens that could impact patient safety.

## CONFLICT OF INTEREST

Cori L. Ofstead and Abigail G. Smart are employed by Ofstead & Associates Inc., an independent company that has received research grants, study materials, educational materials, or consulting contracts from companies involved in endoscope reprocessing or monitoring, including 3M Company, Advanced Sterilization Products, Ambu, Boston Scientific Corporation, Cleanis, Fortive, Healthmark Industries, Pentax, and Steris/Cantel/Medivators. Advanced Sterilization Products has supported our research related to orthophthalaldehyde, human papillomavirus, and reprocessing effectiveness. None of these companies reviewed the submitted work before submission.
